# Effect of Noise on Bornean Orangutans’ Glucocorticoid Metabolite (GCM) Levels

**DOI:** 10.3390/ani15233384

**Published:** 2025-11-22

**Authors:** Marina Bonde de Queiroz, Luiza Figueiredo Passos, Cristiano Schetini de Azevedo, Ivana Schork, Rupert Palme, William J. Davies, Robert John Young

**Affiliations:** 1 School of Science, Engineering and Environment, University of Salford, Manchester M5 4WT, UK; marinabqueiroz10@gmail.com (M.B.d.Q.); w.davies@salford.ac.uk (W.J.D.); r.j.young@salford.ac.uk (R.J.Y.); 2 School of Biological and Environmental Sciences, Liverpool John Moores University, Liverpool L3 5AH, UK; l.figueiredopassos@ljmu.ac.uk; 3 Department of Biodiversity, Evolution and Environment, Universidade Federal de Ouro Preto, Ouro Preto 35400-000, MG, Brazil; 4 Department of Animal and Agriculture, Hartpury University, Gloucester GL19 3BE, UK; ivana.schork@hartpury.ac.uk; 5 Department of Biological Sciences and Pathobiology, Experimental Endocrinology, University of Veterinary Medicine, Veterinärplatz 1, 1210 Vienna, Austria; rupert.palme@vetmeduni.ac.at

**Keywords:** soundscape, stress, orangutan, zoo

## Abstract

Zoos are increasingly hosting out-of-hours events such as ‘Music Nights’ to increase visitation, raising potential animal welfare concerns due to anthropogenic noise pollution. This study examined the physiological stress response, measured through faecal glucocorticoid metabolite (FGCM), of Bornean orangutans (*Pongo pygmaeus*; *n* = 6) at Twycross Zoo during four consecutive weekends of live music events. Faecal samples were collected over these weekends and compared with a comprehensive 24 h acoustic analysis of the enclosure. The acoustic data indicated that the environment was dominated by noise from the ventilation system, which masked the sound from the live music. Comparisons of acoustic metrics between Event and Non-Event periods showed that LAeq, LA10, and LA90 were significantly higher during event hours or open zoo periods. In contrast, daily means did not differ. Group-averaged FGCM concentrations were higher on Event days (mean ± SE: Event = 826 ± 99 ng/g; No Event = 701 ± 44 ng/g), but comparisons for each individual showed no significant differences (Batu: *t* = 0.577, *p* = 0.596; Maliku: *t* = 1.475, *p* = 0.212; Molly: *t* = 0.290, *p* = 0.786; Kibriah: *t* = 0.771, *p* = 0.506). In contrast, FGCM concentrations increased significantly with increasing acoustic levels (LAeq) across individuals, with Batu and Maliku generally showing higher FGCM levels in response to louder days. These findings suggest that the constant background noise may have caused partial habituation. At the same time, individual variation highlights the importance of assessing physiological responses at the individual level, as relying on group-level data may overlook adverse welfare effects on sensitive individuals.

## Simple Summary

Zoos are increasingly hosting concerts outside normal public opening hours to attract additional visitors, raising concerns about the potential stress these loud events may cause for animals, such as the endangered Bornean orangutans. This study aimed to measure the orangutans’ potential physiological stress response while also recording the sound inside their enclosure at Twycross Zoo during four music events. The main finding was that the concert noise did not significantly increase the overall sound level inside the enclosure because a constant, loud ventilation system noise was already dominating the acoustic environment. Individual comparisons of FGCM levels between Event and No Event days for each orangutan also showed no significant differences. However, two individuals, Batu and Maliku, exhibited higher overall FGCM concentrations than the others, and variation in their FGCM levels correlated with increases in acoustic intensity (LAeq). This result highlights that relying on group averages can miss serious negative impacts on sensitive individuals, suggesting that the constant background noise may have led to an adaptive blunting of the stress response in some individuals, while others remained sensitive. Therefore, for better animal welfare, zoos should prioritise understanding the unique needs of each animal and consider practical steps, such as increasing appropriate environmental enrichment on loud days, to help individuals cope with the background noise.

## 1. Introduction

Zoos are increasingly seeking alternative sources of revenue and visitor engagement, such as public events outside regular opening hours, including live music events [1]. Although financially advantageous, these events raise significant welfare concerns due to the potential for increased anthropogenic noise to impact animals [2,3]. High sound pressure levels (SPLs), which quantify the intensity of sound in decibels (dB), can reach 80–100 dB during live music events and may act as stressors, negatively affecting the physiological and behavioural well-being of many captive species [1,2]. Conversely, some studies demonstrate that music at low, ambient SPLs (typically ≤55 dB) can function as beneficial sensory enrichment for animals [3,4]. Sensory enrichment refers to modifications in an animal’s environment that stimulate one or more of its senses, encouraging natural behaviours and enhancing psychological well-being [4].

In zoo settings, the acoustic environment, or soundscape (i.e., the complete range of sounds produced by animals, humans, machinery, vegetation, and the built environment), can undergo significant changes during live events and daily management activities [2]. These changes often involve increased sound pressure levels, especially in the low-frequency range, and greater variability in the overall soundscape [2,5]. Variations in visitor density and behaviour, alterations to husbandry routines (e.g., feeding schedules or access times), shifts in odours or aromas from food stalls, and vibrations from staging or technical equipment can all co-occur with anthropogenic noise, influencing how animals perceive and respond to their environment [6,7,8,9]. Research indicates that increased variability in soundscapes, primarily when dominated by anthropogenic noises, can significantly influence behaviours such as enclosure use, vigilance, vocalisations, and social interactions across various species. These effects have been documented in flamingos [10], mammals [6], primates [2,8], and amphibians [11]. Additionally, zoo soundscapes are influenced not only by visitor numbers but also by husbandry routines and infrastructure noise [8].

Although numerous studies have documented behavioural and some physiological responses to anthropogenic noise in zoo animals, the precise relationship between specific acoustic features and welfare outcomes remains poorly understood [2,6,12]. Assessing the actual impact of acute, high-SPL events may require more than behavioural observation alone, and continuous acoustic monitoring provides a robust method for quantifying these complex sensory environments, identifying dominant noise sources, and tracking temporal patterns [8,13,14,15]. Such monitoring is increasingly recognised as a valuable tool in animal welfare research [16,17], supporting a better understanding of the complete range of sounds animals experience, including those from human activities, machinery, and other animals, as well as those that may not be obvious to human listeners [14,18]. Data gathered through acoustic monitoring can inform decisions for animal management. For example, acoustic monitoring can identify times when external sound sources reach harmful amplitudes, allowing for potential mitigation of exposures [13,14].

Likewise, while behavioural observations provide essential insights into how animals respond to their environment, they may not fully capture internal physiological stress responses. Animals may exhibit reduced behavioural responsiveness while still experiencing persistent physiological stress [19]. Therefore, the measurement of faecal glucocorticoid metabolites (FGCMs) provides a powerful, non-invasive, and complementary tool for understanding how animals cope with stressors in captivity [20,21]. The analysis of FGCMs has been successfully applied to investigate stress related to various zoo operations. These include husbandry changes, construction noise, and fluctuations in visitor numbers across diverse species [12,22,23].

Although some zoos are increasingly hosting public events outside regular opening hours, a conspicuous lack of studies remains that incorporate physiological stress measures, particularly in apes. Furthermore, virtually no research has undertaken a complete 24 h acoustic characterisation of enclosures during such events. While some studies have monitored 24 h zoo soundscapes [5] or compared open versus closed periods [24], these did not specifically investigate structured live music events or other after-hours public activities in conjunction with animal physiological responses. Consequently, a comprehensive understanding of how novel events impact animal welfare is unknown.

Among the species for which understanding welfare implications in dynamic zoo environments is important are the apes. The Bornean orangutan, for instance, is the largest arboreal animal in the world. They are classified as Critically Endangered by the IUCN Red List, with climate change and human pressures being the primary causes of the species’ decline [25]. Currently, approximately 500 individuals are housed in zoos worldwide [26]. These ex situ populations are managed through regional breeding programmes that act as genetically viable insurance populations and contribute to broader conservation initiatives [27,28]. Ensuring the welfare of orangutans in captivity is therefore essential, not only for ethical reasons but also to maintain healthy, behaviourally competent individuals that can support the long-term conservation of the species.

Animals may differ in their sensitivity to anthropogenic noise due to intrinsic characteristics such as age, sex, prior experience, personality traits, genetic makeup, and life history [29,30]. For example, in wild chimpanzees (*Pan troglodytes*), older individuals show a progressive increase in baseline cortisol and a blunting of the diurnal rhythm, independent of changes in dominance rank, indicating age-related dysregulation of the HPA axis [31]. In common marmosets (*Callithris jacchus*), immature males and females respond differently to stressors depending on their developmental stage: sex and age together influence both the magnitude and duration of cortisol responses [32]. In bonobos (*Pan paniscus*), urinary glucocorticoid levels vary across reproductive stages, with mothers showing markedly elevated cortisol during early lactation compared to other phases, demonstrating how reproductive history and physiological state modulate stress responses [33]. Furthermore, studies of hair cortisol in captive and wild primates reveal that both sex and age are significant predictors of long-term glucocorticoid load, emphasising that intrinsic demographic variables must be controlled for in welfare assessments [34]. Recognising such inter-individual variation is therefore fundamental, as relying on average responses may mask substantial differences in how animals cope with environmental stressors, such as noise.

To address this critical need for robust welfare assessments in captive apes and building on previous research into zoo soundscapes and physiological stress in captive animals, this study aimed to assess the impact of live summer music events on the physiological stress response of Bornean orangutans at Twycross Zoo, while simultaneously characterising the complete 24 h acoustic environment of their enclosure. We hypothesised that event days would be associated with higher sound pressure levels within the orangutan enclosure compared to non-event days, and that the orangutans’ FGCM levels would be elevated following these events, reflecting an acute physiological stress response to increased noise. Furthermore, we expected that individual orangutans would vary in their physiological responses, with some showing stronger FGGM elevations than others, emphasising the importance of evaluating individual-level welfare impacts.

## 2. Materials and Methods

### 2.1. Study Site and Subjects

**Figure 1:**
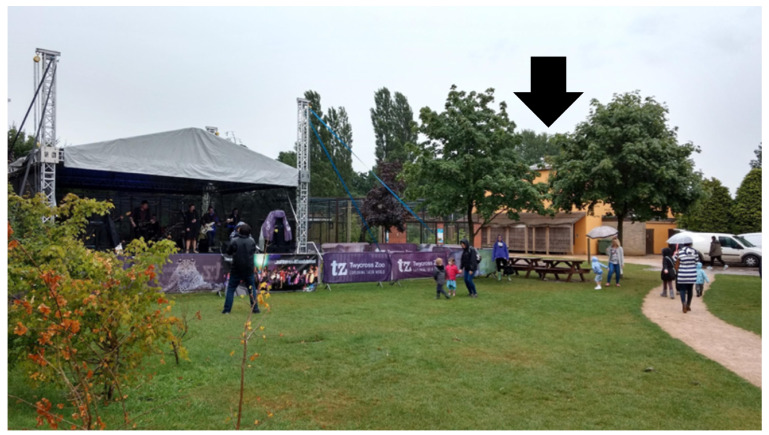
Summer event in Twycross Zoo, UK. Stage concert located next to the orangutans’ enclosure (light-orange building indicated by a black arrow).

The study was conducted at Twycross Zoo, located in Leicestershire, UK, a zoological institution committed to conservation, education, and research. Twycross Zoo participates in a range of ex situ conservation programmes, including the management of Bornean orangutans (*Pongo pygmaeus*) as part of its long-term species conservation strategy, which supports both captive breeding and public education initiatives [35]. The zoo hosts an annual summer live music event, open to the public after standard opening hours (5:00–8:30 p.m.), over four consecutive weekends. These events feature amplified live music (pop-rock) performances on a stage situated near the orangutan enclosure (Figure 1), which may introduce additional auditory and environmental stimuli. The orangutans’ enclosure comprises both indoor and outdoor areas, allowing the animals unrestricted access between spaces throughout the day and during concert events. Routine husbandry, feeding schedules, and visitor access remained consistent between concert and non-concert days.

During this study, Twycross Zoo housed six individuals: an adult male named Batu (DOB: 25 May 1989); an adult female named Kibriah (DOB: 23 January 1977) with an infant (undetermined sex; DOB: 16 June 2017); an adult female named Maliku (DOB: 10 June 1994) with a male infant (DOB: 27 March 2017); and a juvenile female named Molly (DOB: 24 January 2011). The infants were not included in the present study.

### 2.2. Data Collection

The orangutans’ hormone response to noise was studied over four consecutive weekends during the “Summer Sundown” events at Twycross Zoo in July 2017. Individual faecal samples (*n* = 51) were collected by the ape keepers each Friday, Saturday, Sunday, and Monday (always in the morning around 8:00 am), representing the FGCM responses from the previous Thursday, Friday, Saturday, and Sunday [21,36]. However, due to logistical and management reasons, not all animals had samples collected on every designated day. To help keepers identify samples from different animals, edible glitter mixed with the regular feed was used, with each individual receiving a distinct colour [37]. Moreover, because the orangutans’ enclosure was constantly affected by background noise from ventilation and heating systems, and the zoo remained open to visitors year-round, it was not possible to collect samples during a truly low-noise or zero-exposure period. Therefore, non-event days served as the baseline, reflecting typical acoustic and management conditions. This approach enabled the assessment of changes in FGCM concentrations specifically associated with noise increases resulting from events.

Faecal samples were individually stored in labelled hermetic plastic bags and immediately frozen at −20 °C for later FGCM extraction [21]. Therefore, the FGCM levels were collected to compare event and non-event days only.

### 2.3. Analysis of FGCMs

Using the methanol-based protocol [38], a 0.5 g portion of each well-homogenised sample was extracted. 5 mL of 80% methanol was added and shaken in a multivortex (Scientific Industries, New York, NY, USA) for approximately 1.5 min. After centrifugation for 15 min, aliquots (0.5 mL in duplicates) were dried and sent to the University of Veterinary Medicine, Vienna, Austria, for measurement of FGCMs using an 11ß-hydroxyaetiocholanolone enzyme immunoassay (EIA; see [39], which has been successfully validated for orangutans [15]). Species-specific validations have demonstrated that FGCM measurement is suitable for monitoring adrenocortical activity in *Pongo* spp. [30,40]. Glucocorticoid metabolites are measured in nanograms per gram of faeces (ng/g).

### 2.4. Acoustic Metrics

Sound data were collected using a sound level metre (SLM) (Svantek SVAN 957; Svantek, Warsaw, Poland) and a passive acoustic recorder (Wildlife Acoustics Song Metre SM3; Wildlife Acoustics, Maynard, MA, USA) installed inside the enclosure. Both devices measured and recorded the sound continuously for 24 h a day from Thursday to Monday. The SLM was programmed to record sound pressure levels with a 30 s integration period. It was calibrated before and after the measurement period using the calibrator included in the SVAN 957 kit (Svantek, Warsaw, Poland). This specific metre model allows for the simultaneous recording of multiple acoustic metrics, including Leq, Ln, and all required sound level weighting filters (A, C, and Z) within the 1/3-octave frequency bands. This broad range of acoustic metrics was used in the statistical analysis, enabling the selection of the most appropriate metric to explain the species’ responses to noise. The passive acoustic recorder was utilised to identify the source of noise when a simple decibel measurement was insufficient.

### 2.5. Statistical Analysis

All analyses were conducted in R, version 4.5.2 [41]. Daily sound levels collected during the zoo’s operating hours (10:00–20:30) were logarithmically averaged to produce key environmental noise indicators: the equivalent continuous sound level (LAeq, open zoo), the maximum noise level exceeded for 10% of the measurement period (LA10, open zoo), and the noise level exceeded for 90% of the time; that is, without the peaks (LA90, open zoo). In line with standard acoustical practices, LA10 reflects intermittent or peak noise events, while LA90 represents the typical background noise [42]. Equivalent sound levels with an A-weighting response were chosen because orangutans belong to a primate group phylogenetically close to humans, with a hearing system that is anatomically similar, including external, middle, and inner ear structures [43]. Although direct audiometric data for *Pongo* spp. are unavailable, behavioural and anatomical studies of apes and other non-human primates suggest hearing-threshold curves and ear-system structures similar to humans in the main mid-frequency ranges (c. 1–8 kHz) [44,45,46]. Therefore, A-weighting provides a practical and evidence-based method for estimating perceived loudness in these animals, while acknowledging that species-specific audiograms for orangutans are lacking.

All variables were initially explored descriptively, and faecal glucocorticoid metabolite (FGCM) concentrations were analysed in relation to acoustic metrics corresponding to event and non-event periods. Normality of both acoustic and FGCM data was assessed using the Shapiro–Wilk test, which indicated deviations from normality in most variables. Non-parametric Mann–Whitney/Wilcoxon tests were applied for pairwise comparisons between event and non-event periods for LAeq, LA10, and LA90. Boxplots for these metrics were generated using ggplot2 [47] and arranged with patchwork [42] to visualise distributions and highlight differences between event and non-event periods. These analyses quantified relative increases in noise levels during events and identified the acoustic metrics most strongly associated with events [47,48,49].

Given the repeated-measures design, generalised linear mixed models (GLMMs) were fitted using the glmmTMB package [50], with Individual included as a random effect to account for variation across orangutans. Acoustic predictors were initially included as fixed effects; however, due to high collinearity among LAeq, LA10, and LA90, LAeq measured during event periods (LAeq_Event) was retained as the primary predictor following backward elimination. FGCM concentrations were modelled using a negative binomial distribution (nbinom2) to account for overdispersion. Residual diagnostics were checked with the DHARMa package v 0.4.7, and post hoc trends and estimated marginal means were assessed using emmeans and ggeffects [51,52,53].

This approach allowed for the assessment of individual-level variation (i.e., response) and the effect of LAeq_Event on FGCM concentrations. Individual responses to event and non-event periods were specifically examined, reflecting that welfare and stress responses may be individual-specific [46] and not fully captured by group-level analyses. Individual responses were assessed using Shapiro–Wilk tests for normality. Since the data were normally distributed, independent *t*-tests were used to compare FGCM concentrations between the Event (Saturday) and No event (other days) periods. This analysis complements the visual inspection of boxplots, providing a quantitative assessment of individual-level responses.

## 3. Results

**Figure 2:**
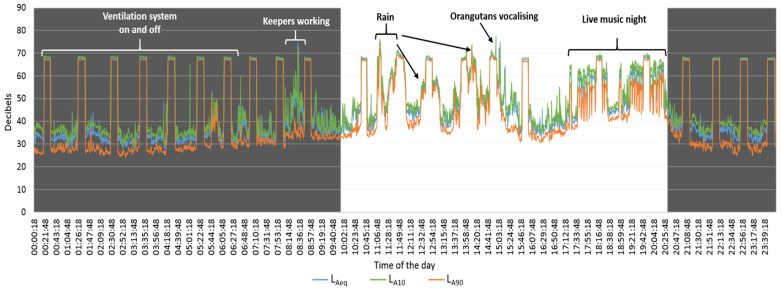
Borneo orangutans’ enclosure sound pressure levels during a summer event day in Twycross Zoo, UK. Continuous 24 h recordings are presented to illustrate the overall daily soundscape. Hours when the zoo was open (white background, 10:00–20:30) indicate the period used for statistical analyses; hours when the zoo was closed are shaded in grey.

**Figure 3:**
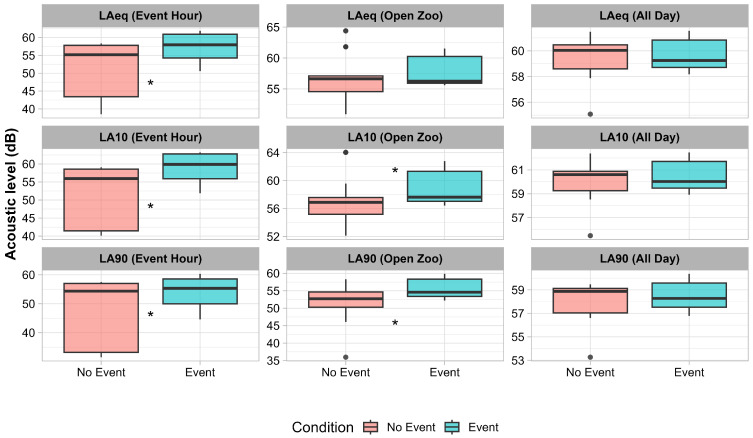
Equivalent sound levels (Laeq) recorded at the Borneo orangutan enclosure in Twycross Zoo (UK). Boxplots show the distribution of acoustic metrics on non-event (No Event; red) and event (Event; blue) days for LAeq, LA10, and LA90. In each plot, the central line represents the median, the box corresponds to the interquartile range (IQR), the whiskers extend to 1.5 × IQR, and points beyond the whiskers indicate statistical outliers. Asterisks between boxplots indicate statistically significant differences between conditions (Wilcoxon test, adjusted *p* < 0.05).

The ventilation system overwhelmingly dominated the acoustic environment of the orangutan enclosure. Even during live music performances, sound pressure levels from the music did not surpass those produced by the ventilation system (Figure 2). Comparisons of acoustic metrics between event and non-event periods revealed that sound levels were generally higher during Event periods, particularly during visitor hours and the open zoo period. During event hours, LAeq and LA10 were significantly higher on event days compared to non-event days (LAeq_EventHour: 59.2 ± 1.23 dB vs. 52.7 ± 1.36 dB, Wilcoxon V = 388, *p*.adj = 0.0066; LA10_EventHour: 59.2 ± 1.23 dB vs. 52.7 ± 1.36 dB, V = 388, *p*.adj = 0.0066), while LA90_EventHour was also higher (54.3 ± 1.63 dB vs. 49.2 ± 1.91 dB, V = 340, *p*.adj = 0.041). During open zoo periods, LA10 and LA90 were significantly elevated on event days (LA10_OpenZoo: 59.1 ± 0.67 dB vs. 57.0 ± 0.62 dB, V = 348, *p*.adj = 0.031; LA90_OpenZoo: 55.8 ± 0.78 dB vs. 51.8 ± 0.96 dB, V = 360, *p*.adj = 0.019), whereas LAeq_OpenZoo showed no significant difference (58.0 ± 0.65 dB vs. 56.8 ± 0.69 dB, V = 279, *p*.adj = 0.567). Considering the entire day, differences between event and non-event periods were not statistically significant for any metric (LAeq_AllDay: 59.7 ± 0.34 dB vs. 59.6 ± 0.28 dB, *p*.adj = 0.991; LA10_AllDay: 60.6 ± 0.36 dB vs. 60.1 ± 0.29 dB, *p*.adj = 0.606; LA90_AllDay: 58.5 ± 0.35 dB vs. 58.1 ± 0.29 dB, *p*.adj = 0.972). These results indicate that increases in acoustic levels were most pronounced during visitor presence and specific events, while overall daily averages remained similar between event and non-event days (Figure 3).

Group-averaged FGCM concentrations were higher on event days compared to non-event days (mean ± SE: Event = 826 ± 99 ng/g, No Event = 701 ± 44 ng/g). However, comparisons of FGCM levels between Event and No Event days for each individual showed no significant differences (Batu: *t* = 0.58, *p* = 0.596; Maliku: *t* = 1.48, *p* = 0.212; Molly: *t* = 0.29, *p* = 0.786; Kibriah: *t* = 0.77, *p* = 0.506). These results are consistent with the GLMM analysis (Table 1), which showed no significant effect of Event on FGCM levels. In contrast, FGCM concentrations increased significantly with increasing acoustic levels (LAeq) across all individuals (Table 1), and the interaction between LAeq and Condition was non-significant, indicating that the positive relationship between acoustic exposure and adrenocortical activity was consistent across both Event and No Event days.

**Figure 4:**
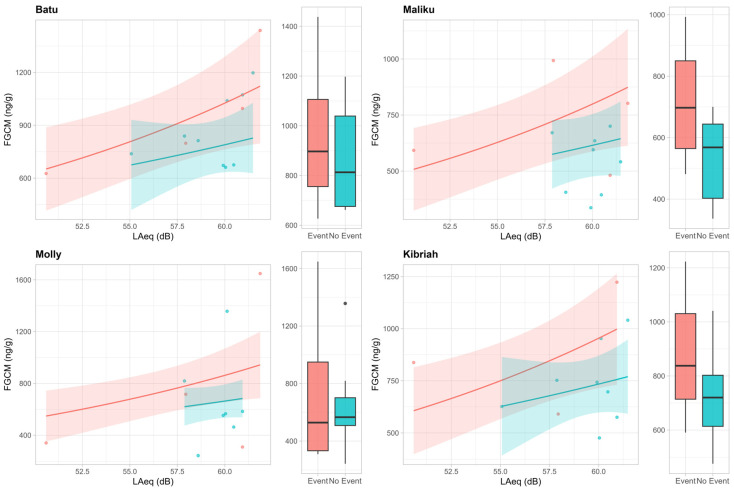
Orangutans’ faecal glucocorticoid metabolite (FGCM ng/g) concentrations in relation to acoustic levels (LAeq) on non-event (green) and event (red) days at Twycross Zoo, UK. Points represent individual observations, lines indicate model-predicted trends, shaded ribbons show 95% confidence intervals, and lateral boxplots display the distribution of FGCM per condition (Event × No event).

Post hoc pairwise comparisons between individuals showed a significant difference in FGCM concentrations between Batu and Maliku (estimate = 0.41. z = 3.09. *p* = 0.011), reflecting individual variability in stress responses. Mean FGCM concentrations ± SE for this comparison were: Batu Event = 964 ± 175 ng/g, No Event = 857 ± 66 ng/g; Maliku Event = 717 ± 113 ng/g, No Event = 535 ± 49 ng/g. All other comparisons between individuals were not significant (all *p* > 0.12) (Figure 4).

## 4. Discussion

Acoustic analysis of the orangutan enclosure at Twycross Zoo revealed that summer music events did increase acoustic energy levels during visitor and event hours. Nevertheless, these increases were small and did not exceed the amplitude of baseline noise generated by the ventilation system, which remained the dominant source of sound throughout the day. Although LAeq and LA10 were significantly higher during event hours, and LA10 and LA90 increased during open-zoo periods, overall daily means did not differ between event and non-event days, indicating that the enclosure’s soundscape is largely buffered against short-term fluctuations in external noise. At a group level, we found no significant differences in FGCM levels between days with and without events for the orangutans involved in this study. The GLMM confirmed that the “Event vs. No Event” comparison did not predict FGCM concentrations. However, it is essential to note that the absence of a group-level response does not imply a uniform pattern across individuals. Instead, our results showed that FGCM concentrations increased significantly with rising LAeq, regardless of day type, indicating that acoustic exposure itself, rather than the occurrence of an event, is the relevant driver of adrenocortical activity. Nevertheless, two individuals exhibited increasing FGCM levels in association with rising sound energy levels, highlighting individual variability in sensitivity to acoustic conditions.

During the event days, faecal glucocorticoid metabolite (FGCM) concentrations were elevated compared to non-event days, although the difference was not statistically significant. This trend aligns with other research that documents elevated FGCM levels in animals exposed to public or anthropogenic events [54]. However, Shapiro–Wilk tests and independent t-tests conducted for each individual (Batu, Maliku, Molly, and Kibriah) confirmed that FGCM concentrations did not differ significantly between Event and No Event periods, reinforcing the GLMM results. In contrast, LAeq had a significant positive effect, indicating that acoustic intensity (i.e., total sound energy), rather than the presence of an event, is the relevant predictor of adrenocortical activity in this context.

Given that overall daily SPLs did not differ between event and non-event days, and that the ventilation system consistently dominated the enclosure’s acoustic environment, the modest increases in noise during events may have been insufficient to trigger a distinct endocrine response. One further consideration is the species’ perceptual sound discrimination. In many species, there is a just-noticeable difference (JND) in intensity below which changes in sound level are not easily perceived. In non-human primates (Japanese macaques *Macaca fuscata*), for example, studies have found intensity discrimination thresholds (difference limens) on the order of ~1.5–2.0 dB for increments, overlapping with human thresholds under some conditions [55]. This suggests that relatively small SPL increases might be below the perceptual noticeability threshold for some individuals. Habitual exposure to the constant ventilation noise may therefore lead to habituation or attenuated HPA-axis reactivity, blunting an acute stress response even when event-related noise increases. Alternatively, the variation observed among individuals could reflect genuine differences in stress perception, with some orangutans experiencing minimal or no stress from the sound events. Such physiological modulation aligns with theories of chronic stress-induced hypocortisolism, acclimation (decreased HPA responsiveness following repeated exposure to a persistent stressor), or facilitation (increased sensitivity to a new stressor after acclimation to another persistent stressor) [56,57]. Recent research further indicates that anthropogenic noise exposure can impact endocrine, cellular, and behavioural systems, altering patterns of glucocorticoid regulation and related physiological pathways [58,59,60,61,62,63], which may help explain why increases in LAeq, not the occurrence of events, were associated with FGCM variability in our study.

It is also essential to recognise that sound levels alone may not fully explain the physiological responses of orangutans. Concerts and other live events can alter the broader soundscape, but they also influence other environmental and management factors. For instance, a study conducted at Tayto Park (Ireland) reported behavioural changes in several zoo-housed species during a live concert. These changes are attributed not only to amplified sound but also to increased human movement and lighting near the enclosures [2]. Similarly, a study conducted at Melbourne Zoo (Australia) found that captive Fiordland penguins (*Eudyptes pachyrhynchus*) and collared peccaries (*Pecari tajacu*) exhibited modified behaviour during musical events, likely influenced by both acoustic exposure and vibrations from sound equipment [12] (this was something we did not measure). Some researchers have observed that evening Christmas events at Knowsley Safari (UK) influence ambient noise patterns, visitor flow, and staff routines, all of which affect animal activity [3]. Furthermore, performance events at Auckland Zoo (New Zealand) have altered the sensory environment for both animals and visitors, demonstrating that zoo soundscapes are dynamic and multisensory settings where odours, lighting, and human presence covary with sound [64]. Collectively, these studies indicate that co-occurring factors, such as vibrations, changes in illumination, odours from food stalls, and modified feeding or cleaning schedules, can influence how animals perceive and respond to their environment, potentially confounding the specific effects of noise. In our study, however, the dominance of the ventilation system limited the degree to which concerts altered the enclosure’s acoustic environment, suggesting that additional sensory stimuli associated with events may also have been dampened or less relevant for the orangutans. At Twycross Zoo, no modifications to routine animal management were made during the events. Although a straw barrier blocked the orangutans’ line of sight to the stage, it did not completely obstruct the sound. Crucially, despite the stage being located less than 50 m from the enclosure and allowing visitor access nearby, these event-related factors did not translate into measurable physiological differences between event and non-event days, reinforcing the conclusion that LAeq, not the event condition itself, was the primary correlate of FGCM variation in this population.

Although there was no group effect, two orangutans (Batu and Maliku) showed differences in FGCM concentrations, and post hoc comparisons confirmed significant variation between these individuals, suggesting that individual differences, rather than group patterns, may better reflect the welfare impacts of environmental noise. Moreover, the GLMM indicated that FGCM levels increased with rising LAeq across all individuals, reinforcing the role of acoustic exposure rather than event occurrence as the relevant predictor. Some orangutans might remain unaffected by events that others find mildly stressful, emphasising the importance of considering individual responses in welfare assessments. Previous research has similarly found that conspecifics may show markedly different physiological or behavioural responses to the same acoustic stressor [65]. Studies have shown that sex and age influence cortisol metabolite responses to noise in European mink (*Mustela lutreola*) [66]. In our study, the absence of a consistent response across orangutans suggests genuine individual differences in sensitivity to acoustic conditions, even when overall noise patterns are similar. This individual variation in FGCM response to loud noises was also observed in other zoo-housed species. For example, Powell et al. [67] reported that giant pandas (*Ailuropoda melanoleuca*) require varying amounts of time for their FGCM to return to baseline levels after exposure to increased stimuli. They noted that individuals exhibit different regulatory mechanisms, leading to diverse stress responses. Additionally, Owen et al. [68] observed similar variations in corticoid responses in giant pandas and suggested that differences in age and perception of environmental noise might explain these variable responses. Furthermore, Clark et al. [69] also found different behavioural expressions to the same stimulus in a group of gorillas (*Gorilla gorilla*). Individual differences are expected due to variations in characteristics that can influence animals’ sensitivity to noise. For instance, Cronin et al. [70] discussed how habituation to specific situations could account for different behavioural reactions to noisy events. Together, these findings highlight the importance of recognising individual traits and coping styles when assessing the welfare impacts of environmental noise, particularly in species with pronounced personality differences such as orangutans.

Studies in humans have shown that individuals differ in their sensitivity to noise, with some being consistently more disturbed than others in the same environment (for a review, see [71]). Animal welfare is similarly regarded as an individual experience, which is why measuring welfare at the group or population level is usually not recommended [72]. Our study suggests that some orangutans were more sensitive to acoustic exposure than others, echoing patterns seen in humans. In particular, Batu and Maliku exhibited higher FGCM concentrations overall. They differed significantly from one another in post hoc comparisons, while the GLMM confirmed that LAeq, but not the occurrence of events, predicted hormonal variation. Although the reasons why these individuals were more strongly affected remain speculative, this finding highlights the risk of overlooking individual variation when responses are grouped. To assess stress responses, we used faecal glucocorticoid metabolites, a non-invasive technique [73]. However, FGCMs may be limited in their capacity to capture short-term responses or slight increases in hormone levels, as their concentrations reflect a cumulative physiological response from approximately 20 h earlier, depending on the species and sampling frequency [21,74]. Because the music performances lasted only a few hours and event-related increases in noise occurred primarily during visitor hours, any acute endocrine response may have been diluted within the broader temporal integration window of FGCMs. Furthermore, while FGCMs offer a robust and non-invasive measure of physiological stress, interpretation can be limited by sample timing, gut transit variability, and the time lag between hormone secretion and excretion. In our dataset, the sampling frequency did not allow for the calculation of individual baselines or fine-scale temporal dynamics, and future studies with more frequent sampling would be required to capture individual responses and event-related hormonal fluctuations more accurately. More frequent and longitudinal sampling could improve temporal resolution and better identify stressor-related fluctuations. Urine sampling could also provide a more immediate measure of physiological responses, as samples collected shortly after the event would better reflect recent hormonal changes [72]. However, this approach is not entirely non-invasive, since keepers would need to enter the enclosure at unpredictable times, potentially causing additional disturbance.

Even without the anticipated outcome of the investigated conditions (night events), our finding that FGCM levels increased with higher LAeq, regardless of whether a music event was occurring, provides valuable insight for zoo management. Identifying potential stress sources for animals in human care is the first step toward achieving good welfare. Although research into zoo soundscapes and the effects of sound exposure mitigation remains limited, some authors have emphasised the importance of understanding the animals’ sensory environment and have proposed that environmental modifications such as acoustic isolation materials, changes in management practices, and even enrichment could help reduce stress caused by noise and support the enhancement of animal welfare related to noise exposure [13,75]. As physical modifications to the enclosures, such as enhanced acoustic insulation, depend on resource allocation and construction logistics, our results suggest that reducing exposure to elevated background noise, particularly that produced by mechanical systems such as ventilation, may be as important as mitigating event-related sound. Twycross Zoo could also evaluate the use of indoor-appropriate environmental enrichment strategies known to buffer or distract from acoustic stressors. These may include providing access to the more acoustically insulated indoor area during noisier periods and increasing the availability of complex foraging devices (e.g., puzzle-feeders, browse bundles, or dispersed foraging opportunities), as well as adding structural enrichment such as additional platforms, nets, or refuge areas. Multi-sensory enrichment, particularly olfactory or tactile stimuli, could further help maintain engagement and reduce the impact of external noise.

While this study focused on the orangutan enclosure at Twycross Zoo, the findings have broader implications for captive animal management in other zoological institutions. The observed individual variation in FGCM responses highlights the importance of considering both group- and individual-level welfare indicators when assessing the impact of sound and related environmental changes. Live events, concerts, or other atypical stimuli can simultaneously alter multiple aspects of the animal’s environment, including acoustic conditions, lighting, human activity, odours, and feeding schedules, potentially complicating interpretations if only sound pressure levels are considered. In our case, the dominance of the ventilation system illustrates how mechanical noise may shape the animals’ acoustic environment more strongly than intermittent events. Notably, the ventilation noise was not constant but exhibited a cyclic pattern, switching on and off for a few minutes each hour; during the live events, this cycle appeared to lengthen, occurring approximately every two hours. This observation underscores the need for continuous rather than event-specific monitoring to fully characterise the acoustic environment. Therefore, monitoring multisensory zoo soundscapes and implementing targeted enrichment or mitigation strategies could be valuable for all zoos aiming to support the welfare of sensitive or noise-vulnerable species.

## 5. Conclusions

This study of the orangutans at Twycross Zoo found that summer night events did not significantly increase environmental sound pressure levels (SPLs), with the ventilation system remaining the primary source of noise and likely masking the concert sounds. Moreover, individual t-tests confirmed that FGCM concentrations did not differ between Event (Saturday) and No event (other days) periods for any orangutan, further supporting the lack of a direct event effect. Instead, our results demonstrated that LAeq was the only significant predictor of FGCM variation, indicating that overall acoustic intensity, rather than the occurrence of events, was associated with adrenocortical activity. This non-significant event effect may be due to the constant background noise causing habituation or a physiological dampening of the acute stress response. However, we noted individual variation in physiological reactions, with Batu and Maliku showing increased FGCM levels and differing significantly from one another, emphasising the risk of overlooking individual welfare needs when relying solely on group data, which is a pattern also seen in other zoo species and human studies.

Although there was no group-level effect, the finding that FGCM concentrations increased with rising acoustic levels provides valuable insight into the welfare of captive animals. Recognising noise, especially persistent mechanical noise, as a potential stressor is the first step towards effective management. We recommend that Twycross Zoo explore strategies to minimise exposure to elevated background noise and consider targeted environmental enrichment during periods expected to be louder, even when these do not coincide with events. This focused approach could help orangutans, especially the more sensitive individuals, better cope with potential stressors. These findings and recommendations are also relevant to other zoological institutions and captive animal facilities, highlighting the importance of monitoring both group and individual responses to acoustic and environmental changes, as well as evaluating the multisensory aspects of zoo environments rather than relying solely on event classifications.

## Figures and Tables

**Table 1:** Results of the Generalised Linear Mixed Model evaluating the effects of acoustic levels (LAeq) and visitor events on Bornean orangutans’ faecal glucocorticoid metabolite (FGCM) levels. SE = standard error.

Variable	Estimate	Std. Error	z Value	Pr(>|z|)
Intercept	3.924	1.131	3.470	0.001
Laeq	0.048	0.020	2.461	0.014
Event	0.721	2.503	0.288	0.773
Laeq/Event	−0.016	0.042	−0.388	0.698

## Data Availability

Data is available at Bonde de Queiroz, Marina; Passos, Luiza; Azevedo, Cristiano; Palme, Rupert; Schork, Ivana; Davies, William; Young, Robert (2025). “Effect of noise on Bornean orangutans’ glucocorticoid metabolites (GCM) levels”. Mendeley Data. V1. doi: 10.17632/8kf93hs2cr.1.

## Abbreviations

The following abbreviations are used in this manuscript:

FGCMs	Faecal glucocorticoid metabolites
SPL	Sound pressure level
dB	Decibel
SLM	Sound level metre
DOB	Date of birth
L_eq_	Equivalent continuous sound level
EIA	Enzyme immunoassay
